# Closed-loop optimization of chromatography column sizing strategies in biopharmaceutical manufacture

**DOI:** 10.1002/jctb.4267

**Published:** 2013-12-26

**Authors:** Richard Allmendinger, Ana S Simaria, Richard Turner, Suzanne S Farid

**Affiliations:** a The Advanced Centre for Biochemical Engineering, Department of Biochemical Engineering, University College LondonTorrington Place, London, WC1E 7JE, UK; b MedImmune Limited, Milstein BuildingGranta Park, Cambridge, CB1 6GH, UK

**Keywords:** biopharmaceutical manufacture, antibody purification technologies, downstream processing design, evolutionary computation, closed-loop optimization, process economics

## Abstract

**BACKGROUND:**

This paper considers a real-world optimization problem involving the identification of cost-effective equipment sizing strategies for the sequence of chromatography steps employed to purify biopharmaceuticals. Tackling this problem requires solving a combinatorial optimization problem subject to multiple constraints, uncertain parameters, and time-consuming fitness evaluations.

**RESULTS:**

An industrially-relevant case study is used to illustrate that evolutionary algorithms can identify chromatography sizing strategies with significant improvements in performance criteria related to process cost, time and product waste over the base case. The results demonstrate also that evolutionary algorithms perform best when infeasible solutions are repaired intelligently, the population size is set appropriately, and elitism is combined with a low number of Monte Carlo trials (needed to account for uncertainty). Adopting this setup turns out to be more important for scenarios where less time is available for the purification process. Finally, a data-visualization tool is employed to illustrate how user preferences can be accounted for when it comes to selecting a sizing strategy to be implemented in a real industrial setting.

**CONCLUSION:**

This work demonstrates that closed-loop evolutionary optimization, when tuned properly and combined with a detailed manufacturing cost model, acts as a powerful decisional tool for the identification of cost-effective purification strategies. © 2013 The Authors. Journal of Chemical Technology & Biotechnology published by John Wiley & Sons Ltd on behalf of Society of Chemical Industry.

## INTRODUCTION

**Figure 1:**
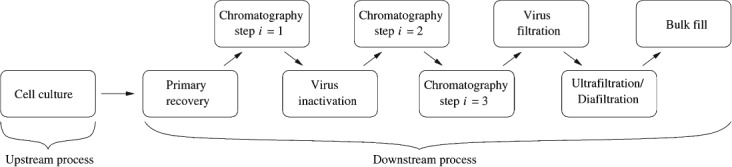
Typical flowsheet for an antibody manufacturing process.

Monoclonal antibodies (mAbs) represent the fastest growing category of therapeutic biopharmaceutical drugs due to their unique binding specificity to targets. Yet, their manufacture is costly and time-consuming. The manufacturing process for mAbs can be divided into two phases (Fig. 1): *upstream processing* (USP) and *downstream processing* (DSP). In USP, mammalian cells expressing the mAb of interest are cultured in bioreactors. Following cell culture, the broth moves to DSP, where the mAb is recovered, purified and cleared from potential viruses using a variety of operations including a number of chromatography steps, such as affinity or ion-exchange chromatography. Chromatography operations are identified as critical steps in a mAb purification process and can represent a significant proportion of the purification material costs (associated, for example, with the use of expensive affinity resins and large amounts of buffer reagents). While alternatives to traditional column chromatography platforms are emerging, industry practitioners are still reluctant to perform major process changes.^1^,^2^ At the same time, it is important to determine how best to use existing production facilities for mAbs.^3^,^4^ This is particularly challenging given the significant improvements in USP productivities that have been accomplished over the past decade with higher mAb concentrations (*titers*) being achieved in cell culture. These improvements have not been matched in purification capacities, leading to concerns over purification bottlenecks and the desire to continuously optimize the design and operation of existing chromatography steps. Hence, to efficiently exploit these cell culture improvements, and account for the growing market for therapeutic mAbs, it has become critical to identify cost-effective purification processes.^1^,^2^,^5^

An approach to tackle these issues, which is also adopted here, is to develop simulation models of mAb manufacturing processes and locate promising chromatography setups using computational methods. For example, Joseph *et al.*^6^ present a simulation model to identify windows of operation for the column diameter, bed height and loading flowrate of a chromatography step using productivity and cost of goods (COG) as performance criteria. A model to find combinations of protein load and loading flowrate that meet yield and throughput constraints has been developed by Chhatre *et al.*^7^ The discrete-event simulation framework proposed by Stonier *et al.*^8^ allows the selection of optimal chromatography column diameters over a range of titers. The methodology used by these authors^6^–^8^ consists of selecting and evaluating specific values within the full range of variation of the critical parameters. However, such an approach may not be feasible for larger decision spaces as considered here, where the number of possible permutations of options means that they can no longer be examined individually. Recently a set of mathematical programming approaches to address chromatography column sizing and sequencing problems were presented by Liu *et al.*^9^,^10^ These methods validated the outcomes of the evolutionary algorithms created in this paper for small problem instances but were found to reach limitations when attempting to solve large problem instances under uncertainty with commercial solvers. This drives the need for more efficient combinatorial optimization methods in this domain.

To address this issue, Simaria *et al.*^11^,^12^ conducted a study on the application of evolutionary algorithms (EAs) to optimize mAb purification processes. EAs represent an example of meta-heuristic combinatorial optimization techniques that function by iteratively evolving a population of candidate solutions using search algorithms based on the principles of natural selection.^13^ Simaria *et al.*^11^,^12^ found that EAs are efficient optimizers in this domain but also recognized that their performance could be improved by further tuning of the EAs. This study conducts a more thorough investigation on this topic. Beginning with a more formal definition of the chromatography equipment sizing problem, this work proposes guidelines on how to tune EAs for this problem and demonstrates the usefulness of certain EA properties when it comes to incorporating user preferences and final decision making.

This study investigates the application of EAs for the identification of *chromatography column sizing strategies* – defined here by the diameter and bed height of a column, the number of columns used in parallel, and the number of cycles a column is run for – that are cost-effective in terms of COG per gram (COG/g) of product manufactured. This task can be formulated as a combinatorial (single-objective) optimization problem subject to multiple constraints and interacting decision variables, uncertain parameters and expensive fitness evaluations (represented by time-consuming computer simulations).

**Figure 2:**
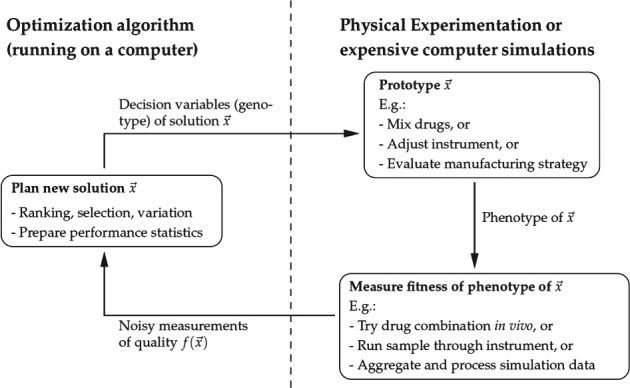
Schematic of closed-loop optimization. The genotype of a candidate solution 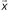 is generated on the computer but its phenotype is experimentally prototyped or alternatively realized by running an expensive computation simulation. The quality or fitness 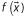 of a solution may be obtained experimentally too and thus may be subject to measurement errors (noise).

The type of problem considered here – where solutions are evaluated by simulation rather than computing some function available in algebraic form – is commonly referred to as a *closed-loop optimization problem*.^14^–^16^ The term *closed-loop* suggests that the setup in such problems establishes an interactive loop between an optimizer and the experimental platform, represented here by the simulator. Figure 2 illustrates the closed-loop setup commonly used in experimental optimization including in this work. Over the years, evolutionary algorithms (EAs) have proven to be efficient, flexible and robust optimizers for a number of closed-loop problems in areas such as shape design optimization, quantum control, drug discovery, analytical biochemistry, fermentation media optimization, marine biosurfactant production, batch distillation design, and biopharmaceutical portfolio management combined with capacity planning.^17^–^27^ For a detailed introduction into the field of closed-loop optimization including challenges, applications, and algorithms employed, please refer to Allmendinger.^14^

An industrially-relevant case study is used to investigate how to tune some of the EA configuration parameters: population size, degree of elitism, number of Monte Carlo trials (needed to cope with uncertain parameters), and constraint-handling method. The fitness landscape of different scenarios of the case study is analyzed also to observe which landscape features pose a particular challenge when optimizing equipment sizing strategies. A comparison between the equipment sizing strategies used by manufacturers (regarded in this paper as the base case) and solutions provided by the EA is presented and a practical procedure based on visual tools for selecting the most preferred sizing strategy is illustrated.

## METHODOLOGY

### Problem domain: chromatography equipment sizing

**Figure 3:**
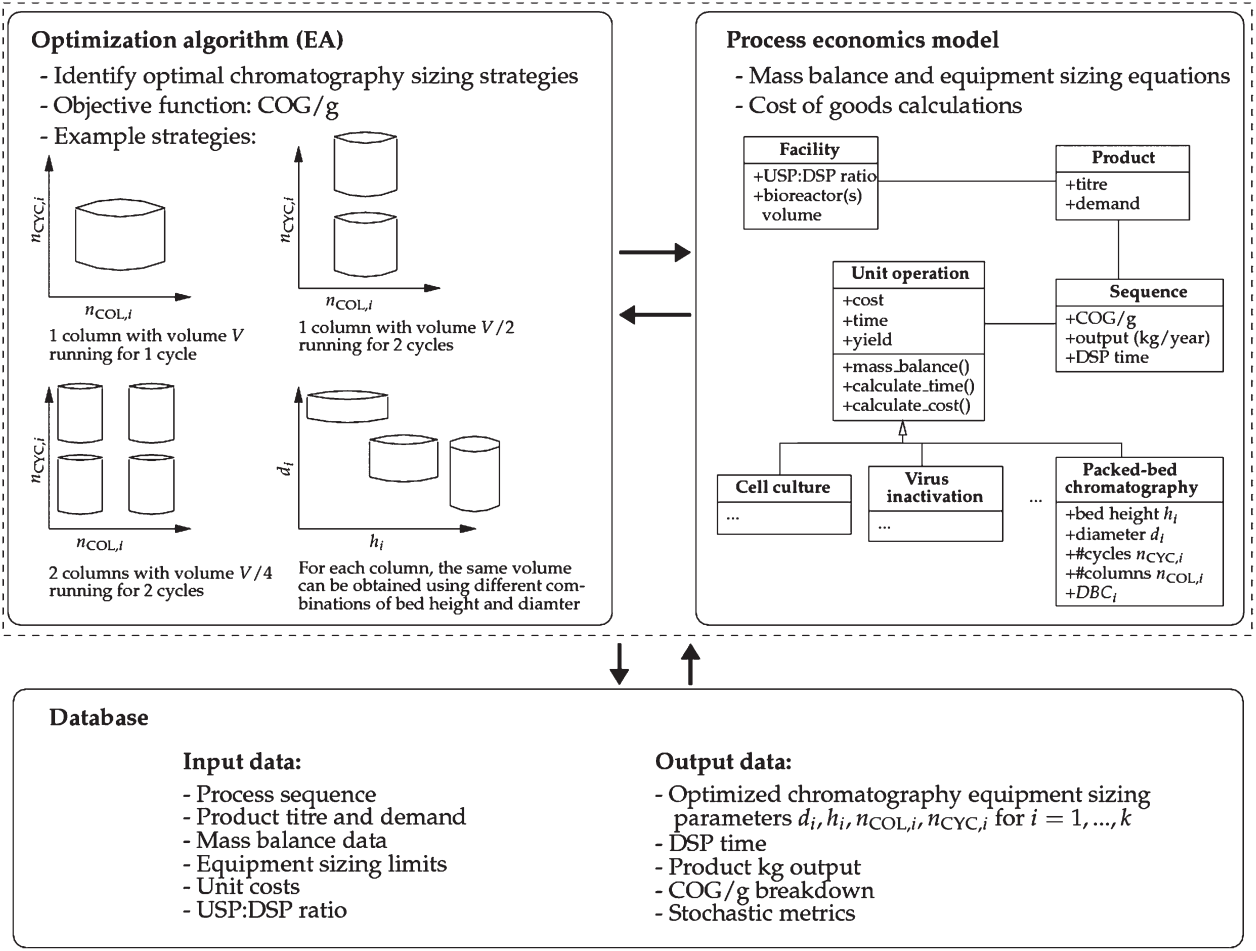
Schematic of the closed-loop platform employed comprising an optimization algorithm (EA) linked to a detailed process economics model; the interaction between the two components is supported by a database. The process economics model (its structure is illustrated using a UML diagram) performs mass balance and cost calculations and the EA determines the best chromatography equipment sizing strategies. A chromatography equipment sizing strategy is defined by the column bed height *h*_*i*_, column diameter *d*_*i*_, number of cycles *n*_CYC,*i*_ and number of columns *n*_COL,*i*_ for each chromatography step  *i* = 1, …, *k* employed. Different sizing strategies may yield the same overall column volume but nevertheless vary in the objective COG/g.

The chromatography equipment sizing problem can be represented as a combinatorial optimization problem with the task of finding the most cost-effective chromatography sizing setup for a sequence of chromatography steps used in the purification process of mAbs. A typical mAb manufacturing process, as shown in Fig. 1, consists of a number of packed-bed chromatography steps, with the chromatography sizing strategy selected having a direct impact on metrics such as manufacturing costs and time, and annual product output. To locate effective sizing strategies, the closed-loop setup illustrated in Fig. 3, which links an optimization algorithm with a process economics model, is employed.

In the following the decision variables, objective function, constraints, and uncertain parameters, to which the chromatography equipment sizing problem considered here is subject to, are described.

#### Decision variables

**Figure 4:**
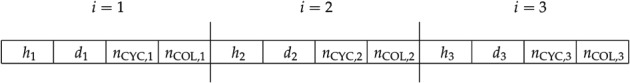
A candidate solution (sizing strategy) with *k* = 3 chromatography steps. Each step *i* = 1, …, *k* is defined by the parameters *h*_*i*_,  *d*_*i*_,  *n*_CYC,*i*_  and *n*_COL,*i*_.

Figure 4 shows the encoding used to represent a solution 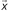 to the chromatography sizing problem. For each chromatography step 1 ≤ *i* ≤ *k* (*k* is the total number of steps) four discrete decision variables were defined related to the sizing and operation of chromatography columns: bed height *h*_*i*_ and diameter *d*_*i*_ of columns, number of cycles *n*_CYC,*i*_ each column is used, and the number of columns *n*_COL,*i*_ operating in parallel. That is, the problem is subject to *k* × 4 discrete variables in total. This captures the trade-offs of using large columns with a single cycle versus smaller columns with multiple cycles as illustrated in Fig. 3. Small changes in bed height were accommodated to account for typical ranges seen in industrial applications and it was assumed that such changes would not affect product quality or recovery. The use of multiple parallel columns per step was also incorporated so as to determine whether this offered significant advantages that might outweigh current preferences to avoid parallel columns due to validation burdens. For each step *i*, the variables define the (i) total volume of resin *V*_*i*_ available for the purification of a product at that chromatography step, and the (ii) processing time *T*_*i*_ that the chromatography step takes; both parameters are calculated according to standard mass balance equations as follows:^26^

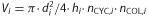
(1)

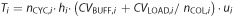
(2)

where *CV*_BUFF,*i*_ and *CV*_LOAD,*i*_ are the number of column volumes of buffer and product load applied per cycle, and *u*_*i*_ is the linear flowrate of the resin used at step *i*.

#### Objective function

The objective *f* is to find a chromatography sizing setup that yields minimal cost of goods per gram (COG/g) of product manufactured. The COG includes both direct (resource) costs (e.g. resin, buffer and labor costs) and indirect costs (e.g. facility dependent overheads, such as maintenance costs and depreciation), and is divided by the total annual product output *P* to yield the metric COG/g. The COG/g values are obtained by running a detailed process economics model, which simulates the different purification steps based on mass balance and cost equations as defined by Farid *et al.*^28^ A more detailed explanation of the COG/g components is presented in Simaria *et al.*^12^ A key feature of this model is the impact of processing time on COG/g. The annual demand *D* is an input of the model and it is used to calculate the bioreactor size assuming a given number of batches is produced annually in the facility. However, if a particular equipment sizing strategy leads to long processing times, it may not be possible to meet the total number of batches and hence the annual product output would be below the production target. This penalizes the objective function through the decrease of the denominator in COG/g (i.e. the total amount of grams of product produced).

#### Constraints

The problem is subject to two types of constraints:

1. Each chromatography step *i* = 1, …, *k* needs to satisfy a resin requirement constraint to ensure that the resin volume *V*_*i*_ available for purification at step *i* is sufficient to process the mass of product *M*_*i*_ entering that step, given the resin's dynamic binding capacity *DBC*_*i*_ and the maximum utilization factor κ. Formally, this constraint can be defined as 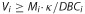
(3)
  Solutions violating this constraint are considered infeasible and handled using one of the constraint-handling strategies introduced later.
2. There is also a demand constraint to ensure that the amount of product manufactured *P* is sufficient to satisfy the annual demand *D*, or *P* ≥ *D*. This constraint may be violated for column sizing strategies with long chromatography processing times *T*_*i*_. Using COG/g as the objective function (recall that the product output *P* is in the denominator of this metric) was found to be sufficient to cope with this constraint.

#### Uncertainties

Uncertainty related to the product titer can have a significant impact on the annual product output *P*. As the equipment sizing is a function of an expected titer value for bioreactors through to chromatography columns, titer fluctuations can cause (i) failure to meet demand (if titer is lower than expected), or (ii) product waste (if titer is higher than expected and equipment capacity is insufficient to process the excess). Other sources of uncertainty (e.g. yield, and processing times) may be present and are realistic but are not considered in this paper.

### Search algorithms

To gain insight into the behaviour of evolutionary search algorithms on the chromatography sizing problem, four types of search algorithms were considered: a simple generational genetic algorithm (SGA), a genetic algorithm with generation gap (GA-GG), a genetic algorithm with a (*μ* + *λ*)-ES reproduction scheme (GA-ES), and a population of stochastic hill-climbers (PHC). Algorithm 1 shows the pseudocode of the search algorithms and the way the constraint handling strategies (which are introduced below) are managed.

**Algorithm 1:**
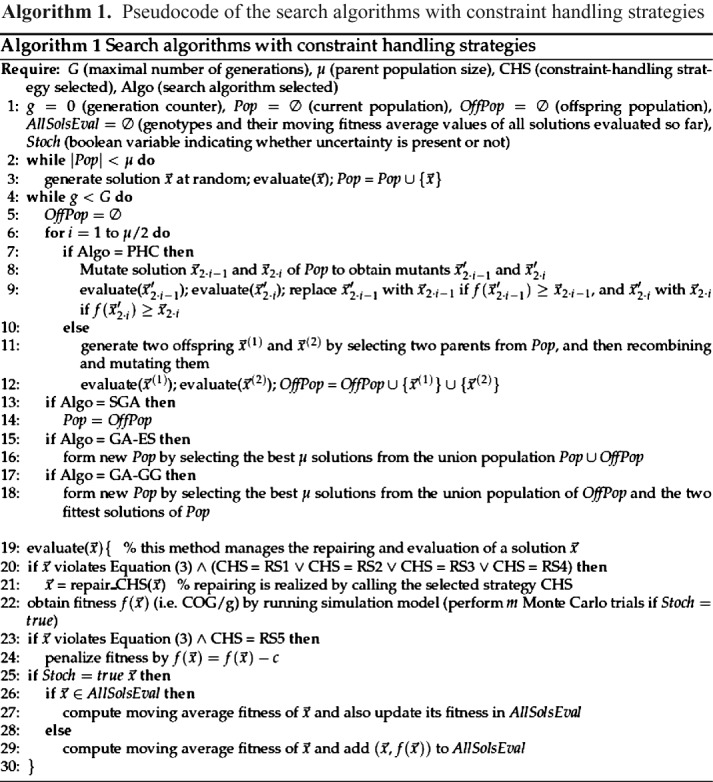
Pseudocode of the search algorithms with constraint handling strategies

All four algorithms began the search with the same initial population containing *μ* randomly generated solutions. The algorithms used also the same mutation operator, which selected a decision variable value at random from the set of possible values. SGA used uniform crossover and random flip mutation as the variation operators, and binary tournament selection (with replacement) for parental selection; for environmental selection, it replaced the entire current population with the offspring population. GA-GG and GA-ES differ from SGA in the environmental selection step only. With GA-GG, the new population was formed by selecting the fittest *μ* solutions from the combined pool of the offspring population and the two fittest solutions of the current population. With GA-ES, a greater degree of elitism was employed and the fittest *μ* solutions from the combined pool of the current population and the offspring population were selected. PHC maintained a population of stochastic hill-climbers, which, at each generation *g*, independently underwent mutation and replaced their parent if it was at least as fit.

#### Accounting for uncertainty

To account for titer variabilities, *m* Monte Carlo trials (based on the titre probability distribution Tr(2.6,3.0,3.4)) were performed for each candidate solution. The fitness of a solution was then the average of the COG/g values across the *m* trials, and this average was updated if a solution happened to be evaluated multiple times during an optimization procedure.

#### Handling of infeasible solutions

Five constraint-handling strategies were analyzed to cope with infeasible solutions (violating Equation 3). Four of them (RS1, RS2, RS3 and RS4) *repaired* infeasible solutions, i.e. modified the genotype of a solution, while strategy RS5 avoided repairing in some way in order to prevent drift-like effects in the search direction (which may occur due to repairing).

The four ‘repairing’ strategies iteratively increased the values of the decision variables (associated with a particular chromatography step *i*), one variable at a time, until Equation 3 was satisfied or until the maximum value of a variable was reached, in which case the value of another variable was increased. The sequence in which the variables were modified affected the search. To investigate this effect, different sequences, represented by the strategies RS1 to RS4, were analyzed. The strategy, RS1, applied repairing according to the decision variable sequence *d*_*i*_ → *n*_CYC,*i*_ → *h*_*i*_ → *n*_COL,*i*_ (where *i* is the chromatography step violating Equation 3); this sequence represents typical rules applied in equipment sizing scale-up models. The strategy, RS2, employed the inverse sequence of RS1, i.e. *n*_COL,*i*_ → *h*_*i*_ → *n*_CYC,*i*_ → *d*_*i*_. The strategies, RS3 and RS4, switch between different repairing sequences during an optimization procedure. While RS3 chooses at random between the two sequences employed by RS1 and RS2, the strategy, RS4, chooses at random among all possible repairing sequences (note, there are 4! different sequences to choose from) whenever it needs to be repaired. The approach employed by RS4 would be plausible, for example, if no prior knowledge about promising repairing sequences was available. The strategy, RS5, does not apply repairing but penalizes infeasible solutions by degrading their fitness by a large fixed penalty value *c*.

The closed-loop platform comprising the search algorithms and process economics model was coded in C# (Microsoft Visual Studio 2010, Microsoft Corporation, WA, USA) which was linked to an input/output database in Microsoft Access (Microsoft Office 2010, Microsoft Corporation, WA, USA).

### Case study description

An industrially-relevant case study was considered to demonstrate the ability of the framework to generate cost-efficient and robust chromatography equipment sizing strategies. The case study focuses on a single-product mAb manufacturing facility that employs a process sequence as shown in Fig. 1 (with *k* = 3 chromatography steps) to satisfy a total product demand of *D* = 500 kg/year with an expected titer of 3 g/L. The main details of the chromatography steps used in the model to perform mass balance and cost calculations are presented in Table1. Titer variabilities were modelled using the triangular probability distribution, Tr(2.6,3.0,3.4). Three scenarios of this case study with different ratios of USP:DSP trains were investigated: 1:1, 2:1 and 4:1. The USP train refers to the number of bioreactors operating (in a staggered mode), and an increase in the USP:DSP ratio corresponds to a decrease in the DSP window, the time available to perform chromatography. The range of possible decision variable values is 15 cm ≤ *h*_*i*_ ≤ 25 cm (11 values), 50 cm ≤ *d*_*i*_ ≤ 200 cm (16 values from a set of commercially available column diameters), 1 ≤ *n*_CYC,*i*_ ≤ 10 (10 values), 1 ≤ *n*_COL,*i*_ ≤ 4 (4 values), *i* = 1, 2, 3; i.e. there are (11 × 16 × 10 × 4)^3^ ≈ 3.5 × 10^11^sizing strategies in total. The equipment sizing strategy of the base case is based on empirical rules used by manufacturers: a single column *n*_COL,*i*_ = 1 with a fixed bed height of *h*_*i*_ = 20 cm is run for a fixed number of cycles *n*_CYC,*i*_ = 5  with the diameter size *d*_*i*_ being calculated such that the resulting total resin volume *V*_*i*_ (calculated according to Equation 1) satisfies the resin requirement constraint (Equation 3). Additionally, an attempt is made to reduce the number of cycles to *n*_CYC,*i*_ = 4 (while keeping the other sizing parameters fixed); this setting is realized if the resulting resin volume still satisfies Equation 3.

**Table 1:** Key input parameters for the *k* = 3 packed-bed chromatography steps in the case study

Input parameters	*i* = 1	*i* = 2	*i* = 3
Dynamic binding capacity (g/L)	35	40	100
Linear velocity (cm/h)	350	200	300
Buffer volume (CV)	35	25	10
Resin price ($/L)	13000	630	1100

Note: CV = number of column volumes.

The experimental study investigated different settings of the parameters involved in the search algorithms. However, if not otherwise stated, the default settings given in Table2 were used. Any results shown are average results across 20 independent algorithm runs. To allow for a fair comparison of the search strategies, a different seed was used for the random number generator for each EA run but the same seeds for all strategies. This allows for the application of a repeated-measures statistical test, the Friedman test,^29^ to investigate performance differences between algorithmic setups.

**Table 2:** Default parameter settings of search algorithms

Parameter	Setting
Parent population size *μ*	80
Offspring population size *λ*	80
Per-variable mutation probability	1 / *l*
Crossover probability	0.6
Constraint-handling strategy	RS1
Number of generations *G*	25
Penalty value *c*	5000
Monte Carlo trials *m*	25

## RESULTS AND DISCUSSION

**Figure 5:**
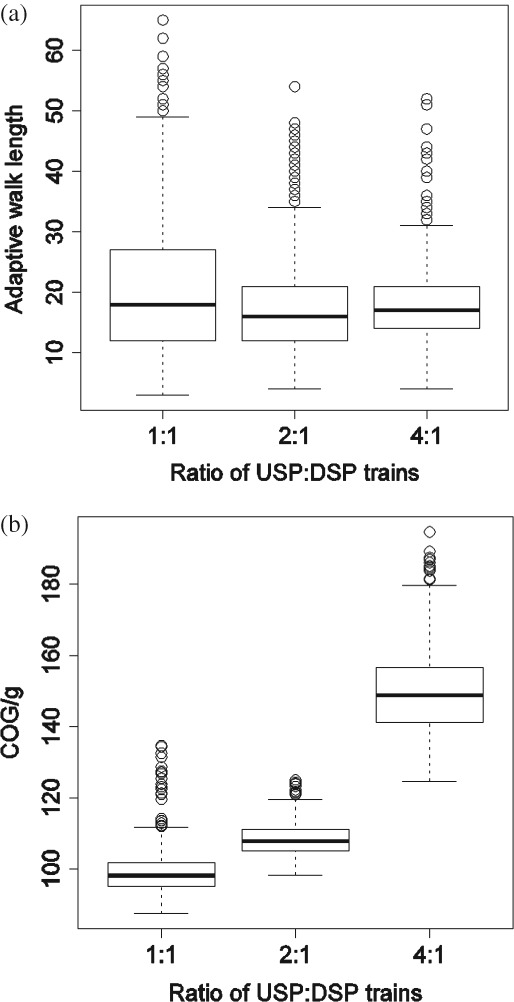
Boxplots showing the distribution of the (a) length and (b) final fitness (COG/g) of 1000 adaptive walks for different USP:DSP ratios. The box represents the 25th and 75th percentile with the median indicated by the dark horizontal lines. The whiskers represent the observations with the lowest and highest value still within Q1-1.5×IQR and Q3+1.5×IQR, respectively; solutions outside this range are indicated as dots. Q1 and Q3 are the 25th and 75th percentile, and the interquartile range is IQR=Q3-Q1.

Before analyzing the behaviour of the search algorithms on the chromatography equipment sizing problem, an indication of the properties of the fitness landscapes spanned by three case study scenarios is given. For this, the adaptive walks method was adopted.^16^,^30^ Starting from a randomly generated solution, an adaptive walk calculates the fitness of all neighbours of the solution that can be generated with a single mutation step, and selects one of the fitter neighbours at random to move to. If there is no fitter neighbour, then the walk has reached a local optimum and terminates. In this study, 1000 adaptive walks (using a fixed titer of 3 g/L) were performed on the landscape of each scenario, and the length of each walk was recorded. Figure 5(a) shows the distribution of the adaptive walk length in the form of boxplots. From the plot it can be observed that increasing the USP:DSP ratio tends to decrease the average length and variability of an adaptive walk. That is, the landscape becomes more rugged, or, equivalently, the number of local optima increases. This pattern is due to tighter DSP windows, which cause more solutions to violate the demand constraint and thus makes the problem harder to solve. This also causes an increase in the COG/g values as indicated in Fig. 5(b). The next section presents an analysis of how the search algorithms fared for both the deterministic (using a fixed titer of 3 g/L) and stochastic scenario.

### Deterministic product titer

**Figure 6:**
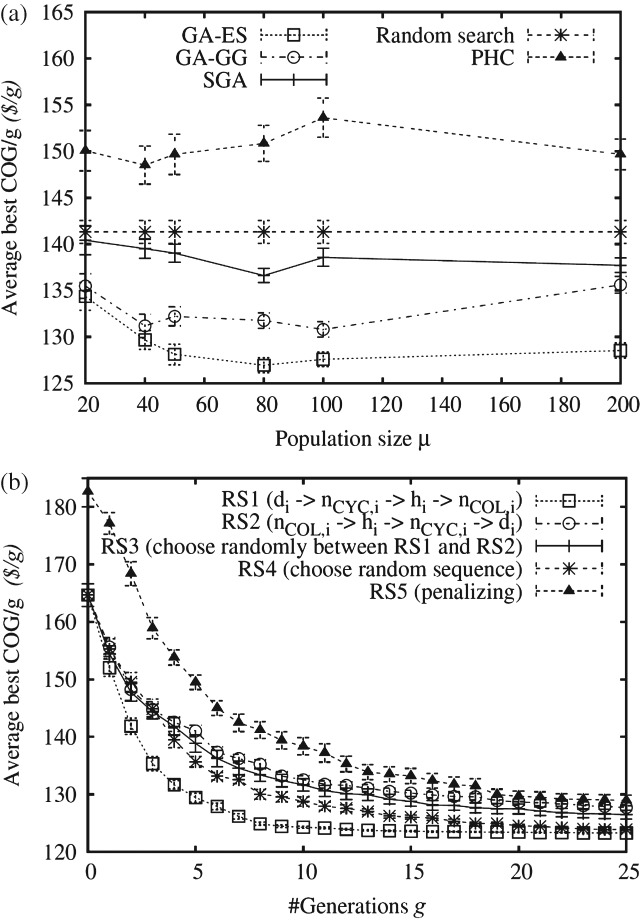
(a) Average best COG/g (and its standard error) obtained by different search algorithms as a function of the population size *μ*; the number of fitness evaluations available for optimization was fixed to #Evals = 2000, i.e. the number of generations is *G* = ⌊2000/*μ*⌋. (b) Average best COG/g, as a function of the generation counter *g*, obtained by GA-ES using different repairing strategies. Both experiments were conducted on a chromatography equipment sizing problem featuring a ratio of USP:DSP trains of 4:1. For each setting shown on the abscissa, a Kruskal–Wallis test (significance level of 5%) has been carried out. In (a), GA-ES performs best for *μ* > 40 while, in (b), RS1 performs best in the range 1 < *g* < 15.

Figure 6(a) analyzes the performance of the search algorithms as a function of the population size *μ*. The aim of this experiment was to understand whether a large population should be evolved for few generations, or a small population for many generations. This understanding is important when optimizing subject to limited resources, such as limited computational power and time constraints. The figure illustrates that: (i) a population size of around 40 ≤ *μ* ≤ 80 yielded the best performance for the GA-based algorithms (SGA, GA-GG and GA-ES); (ii) GA-ES found the most cost-effective strategies; and (iii) random search outperforms PHC. Small population sizes, or search algorithms employing no elitism, such as SGA, did not perform well due to the high probability of getting trapped in one of the many local optima of the fitness landscape. Large population sizes converged slowly due to the low number of generations available for optimization. PHC was inferior to random search because the hill-climbers could get trapped in local optima, in which case further improvements were unlikely, while random search kept on generating (at random) new and potentially fitter solutions. (Note, the performance of random search is constant for different values of *μ* as it depends only on the total number of function evaluations available.)

Figure 6(b) investigates the performance impact of the five constraint-handling strategies, RS1 to RS5, when augmented on GA-ES (a similar performance impact was present for the other search algorithms). From the plot it is apparent that the constraint-handling strategy employed had an effect on the convergence speed and the final solution quality. Furthermore, it indicated that a repairing strategy (RS1, RS2, RS3 and RS4) performed better than a non-repairing strategy (RS5). The superior performance of RS1 is due to the fact that the variable *d_i_* is modified (increased) first when repairing a solution. Unlike to the other variables, an increase in di is often sufficient to just satisfy the resin requirement constraint without increasing the processing time. From the performance obtained with RS2, RS3 and RS4 it can be concluded that if di cannot be changed, then either the variable *n*_CYC,*i*_ or *h*_*i*_ should be modified to meet the resin requirement constraint.

### Stochastic product titer

**Figure 7:**
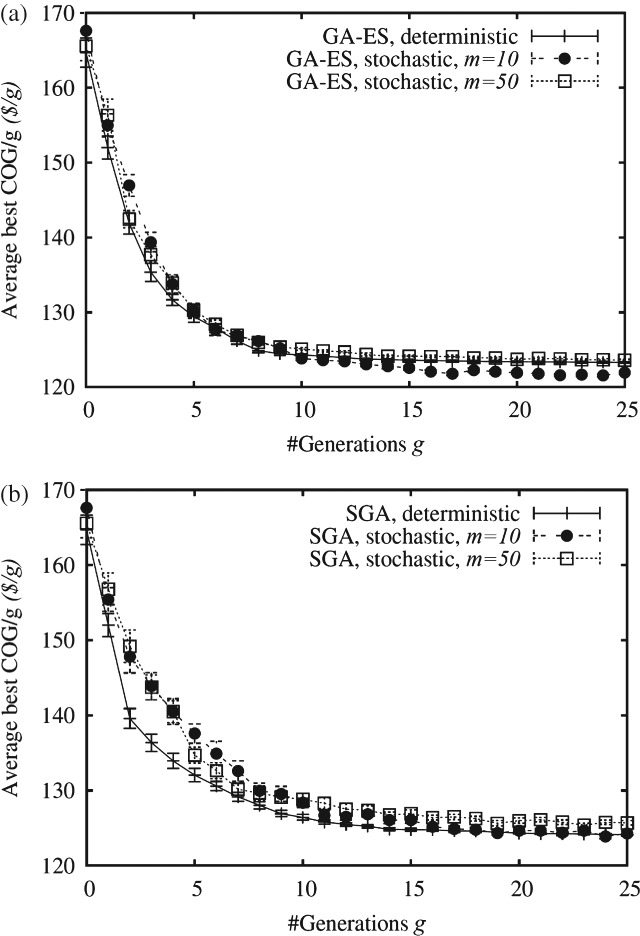
Average best COG/g (and its standard error) obtained by (a) GA-ES and (b) SGA in a deterministic and stochastic environment (using different values for the number of Monte Carlo trials *m*) as a function of the generation counter *g*. For each setting shown on the abscissa, a Kruskal–Wallis test (significance level of 5%) has been carried out. In (a), GA-ES with *m* = 10 performs best for *g* > 15, while, in (b), SGA, deterministic, performs best in the range 1 < *g* < 6.

The performance of the algorithms was then investigated in the presence of uncertain product titers for different values of Monte Carlo trials *m*. Figure 7 indicates that uncertainty impacts negatively the convergence speed and under certain circumstances also the final solution quality. This impact tends to be less severe as the degree of elitism employed by an algorithm increases (i.e. the performance of GA-ES is less affected than that of SGA). Elitism can help circumventing this issue as it causes a population to converge (quickly) to a (local) optimal region and then exploit this region. However, on the other hand, too much elitism (Fig. 7(a)) may disturb and prevent the generation of innovative solutions; here, optimization in a stochastic environment using relatively small values of *m* can yield better performance than optimization in a deterministic environment due to the greater randomness in the search. When the optimizer does not employ elitism (Fig. 7(b)), however, any additional randomness in the search may be a burden (because it can cause a population to oscillate between different regions of the search space, preventing or slowing down convergence towards promising regions).

**Figure 8:**
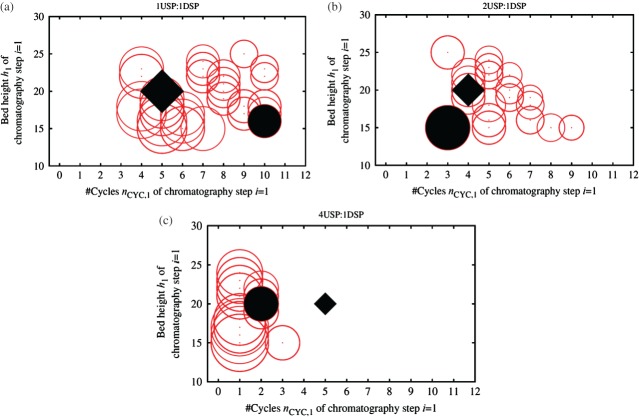
Column sizing strategies for the most expensive chromatography step (*i* = 1) found by GA-ES at the end of the search across 20 independent algorithm runs (within an uncertain optimization environment) (bubbles) for the scenarios (a) 1USP:1DSP, (b) 2USP:1USP and (c) 4USP:1DSP. The size of a bubble is proportional to the variable *d*_1_; all solutions feature the setup *n*_COL,1_ = 1. The COG/g values of all solutions found by the EA for a particular scenario are within 3% of each other. For each scenario, the filled bubble represents the optimal setup found by the EA. The base case setup is indicated with a filled diamond and was not part of the solution set found by the EA for the scenarios 1USP:1DSP and 4USP:1DSP.

In the following, the discussion will be limited to results obtained with GA-ES, as it was the best performing optimizer. The Monte Carlo trial setting *m* = 25 will be used to account for the trade-off between converging reliably to high quality solutions and being able to escape from suboptimal search regions. Figure 8 shows the sizing strategies for the most expensive chromatography step (*i* = 1) found by GA-ES for the USP:DSP ratios 1:1 (Fig. 8(a)), 2:1 (Fig. 8(b)) and 4:1 (Fig. 8(c)) at the end of the search across 20 independent algorithmic runs. The filled bubble in each plot indicates the best solution found by the EA, and the filled diamond the base case setup, which will be discussed in more detail in the next section. From the plots it is apparent that, when moving from 1USP:1DSP to 4USP:1DSP, the number of cycles tends to decrease as the time available for purification shortens. The EA finds more similar solutions for the scenario 4USP:1DSP than for 1USP:1DSP because the problem is harder to solve, as already indicated in the landscape analysis conducted previously.

### Comparison with the base case

**Figure 9:**
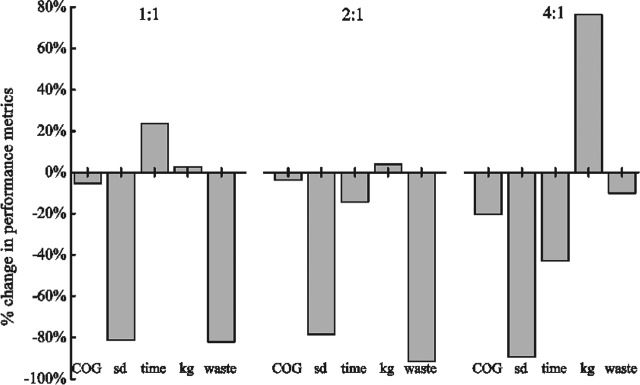
Percentage change in key performance criteria of the best solution found by the stochastic EA relative to the base case for different ratios of USP:DSP trains (COG = average COG/g, sd = standard deviation of COG/g, time = DSP time, kg = average product output, waste = average amount of product wasted due to titer fluctuations). The stochastic values for the base case sizing strategies were obtained by running the same number of Monte Carlo simulations on batch titer as performed for the best solution found by GA-ES.

Figure 9 compares key performance metrics between the best equipment strategy found by GA-EA and the base case setup for chromatography step *i* = 1 (as this was the most expensive step) for the different ratios of USP:DSP trains in a stochastic optimization environment. The bar chart demonstrates that the EA discovered chromatography column sizing strategies that improved the value of the objective function COG/g relative to the empirical approach to column sizing often adopted by industry (i.e. base case, as described earlier) with savings in COG/g of up to 20% being achieved. The characteristics of the optimal solutions found by the EA depend on the type of scenario being addressed. For 1USP:1DSP, where the DSP window is unconstrained at 14 days, the optimized column sizing strategy employs a smaller column running for more cycles than in the base case. This reduces the amount of resin purchased and thus also the COG/g. In the scenario 4USP:1DSP, where the DSP window is constrained to 4 days, the column sizing strategies are optimized to run faster (i.e. fewer cycles and larger diameters), increasing the output kg produced and thus minimizing COG/g.

The use of stochastic EAs permitted the identification of more robust solutions, better equipped to handle titer fluctuations, as shown by the lower standard deviation values of COG/g and reduced product waste (Fig. 9). In the base case approach, the columns are sized according to the expected average titer without specifically accounting for titer fluctuations. Hence, in situations where titers are higher than expected, the columns may not have sufficient excess capacity to cope with higher product loads, leading to product waste. The sizing strategies found by the EA exhibit higher resin volumes and thus overcome this issue to a certain extent.

### Incorporation of user preferences for selection of strategies

**Figure 10:**
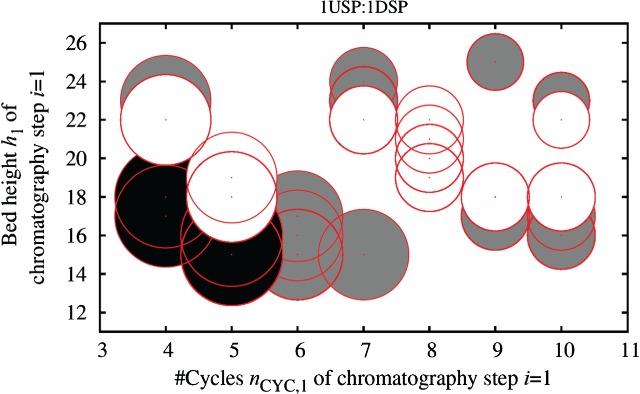
Column sizing strategies for the most expensive chromatography step (*i* = 1) found by GA-ES at the end of the search across 20 independent algorithm runs for the scenario 1USP:1DSP. The size of a bubble is proportional to the column diameter *d*_1_; all solutions feature the setup of 1 column per step, *n*_COL,1_ = 1. Black bubbles: *d*_1_ > 1.6m. Grey bubbles: bed height *h*_1_ > 22 or *h*_1_ < 18 cm, white bubbles: meet both constraints.

For all scenarios, the solutions in the final population found by GA-ES have COG/g values that do not differ by more than 3% of each other, and thus can be considered as valid alternatives available to the decision maker. This allows for the consideration of further criteria and preferences when selecting the strategy to be implemented in the real world, as illustrated in Fig. 10. For example, if space limitations and ease of operation considerations restrict the diameter of columns that can be used to *d*_*i*_ ≤ 1.6 m, then solutions that do not meet the requirements can be excluded (black bubbles in Fig. 10). Also, due to validation issues there might be the need to narrow the range of variation of the column bed height. The grey bubbles in Fig. 10 represent strategies that would not be feasible if the bed height was required to be in the range 18 ≤ *h*_*i*_ ≤ 22 cm. Therefore the white bubbles are the strategies that meet all criteria. The plot illustrates that even with these user preferences, the user still has a set of alternative solutions with similar COG/g values to choose from, thus providing greater flexibility to meet pressures for continuous process improvement.

## CONCLUSIONS

This paper has considered a real-world problem concerned with the identification of cost-effective equipment sizing strategies for purification processes (with focus on chromatography steps) which are core to all bioprocesses. The industrial case study was applied to monoclonal antibodies which represent the fastest growing segment of the pharmaceutical industry where a significant focus is on the need for more cost-effective and robust purification processes for different facility configurations. The application was formulated as a combinatorial closed-loop optimization problem subject to (i) expensive fitness evaluations (relying on time-consuming computer simulations), (ii) multiple dependent decision variables (related to properties of a chromatography column), (iii) constraints (related to resin requirements and product demand targets), and (iv) uncertain parameters (related to variations in product titers, which were accounted for using Monte Carlo simulations).

The investigation highlighted that EAs can identify a diverse set of equipment sizing strategies that are more cost-effective than the strategies used in industry. In particular, the analysis demonstrated that an EA performs best when elitism is employed in combination with a small number of Monte Carlo trials, infeasible solutions are handled using a non-trivial repairing strategy, and (when resources are limited) a medium-sized population (a size between 30 ≤ *μ* ≤ 80) is evolved for a relatively large number of generations. Furthermore, the study illustrated that having available a diverse set of cost-effective sizing strategies (rather than a single one) is beneficial when it comes to account for user preferences and selecting a strategy to be employed in the real world.

Future research will look at extending the equipment sizing problem considered here with relevant decision variables related, for example, to the sequence of a purification process employed and each step's operating conditions. The ultimate goal is to formulate an optimization problem that covers as many relevant aspects arising along the lifecycle of a biopharmaceutical product as possible. The benefit of solving this problem is that decisions regarding the development of a product can be made at an early stage, resulting in potentially significant financial savings.

## Abbreviations

Candidate solution
*f*: Objective function to be optimized
*k*: Total fixed number of chromatography steps in a manufacturing procress
*h*_*i*_: Column bed height at chromatography step *i* (cm)
*d*_*i*_: Column diameter at chromatography step *i* (cm)
*n*_CYC,*i*_: Number of cycles each column at chromatography step *i* is used for
*n*_COL,*i*_: Number of columns operating in parallel at chromatography step *i*
*V*_*i*_: Total volume of resin available at chromatography step *i* (L)
*T*_*i*_: Processing time of chromatography step *i* (h)
*M*_*i*_: Mass of product entering chromatography step *i* (g)
*DBC*_*i*_: Dynamic binding capacity of the resin used at chromatography step *i* (g/L)
*P*: Annual product output (kg)
*D*: Annual demand (kg)
*m*: Number of Monte Carlo trials
*μ*: Parent population size used by the search algorithms
*λ*: Offspring population size used by the search algorithms
*l*: Number of decision variables to be optimized by the search algorithms

## References

[b1] Low D, O'Leary R, Pujar NS (2007). Future of antibody purification. J Chromatogr B.

[b2] Langer E (2009). Downstream factors that will continue to constrain manufacturing through 2013. BioProcess J.

[b3] Kelley B (2007). Very large scale monoclonal antibody purification: the case for conventional unit operations. Biotechnol Prog.

[b4] Kelley B (2009). Industrialization of mab production technology: the bioprocessing industry at a crossroads. MAbs.

[b5] Farid SS, Gottschalk U (2009). Process economic drivers in industrial monoclonal antibody manufacture. Process Scale Purification of Antibodies.

[b6] Joseph JR, Sinclair A, Tichener-Hooker NJ, Zhou Y (2006). A framework for assessing the solutions in chromatographic process design and operation for large-scale manufacture. J Chem Technol Biotechnol.

[b7] Chhatre S, Thillaivinayagalingam P, Francis R, Titchener-Hooker NJ, Newcombe A, Keshavarz-Moore E (2007). Decision-support software for the industrial-scale chromatographic purification of antibodies. Biotechnol Prog.

[b8] Stonier A, Smith M, Hutchinson N, Farid SS (2009). Dynamic simulation framework for design of lean biopharmaceutical manufacturing operations. Comput Aid Chem Eng.

[b9] Liu S, Simaria AS, Farid SS, Papageorgiou LG (2009). Mixed integer optimisation of antibody purification processes. Comput Aid Chem Eng.

[b10] Liu S, Simaria AS, Farid SS, Papageorgiou LG (2013). Designing cost-effective biopharmaceutical facilities using mixed-integer optimization. Biotechnol Prog.

[b11] Simaria AS, Gao Y, Turner R, Farid SS (2011). Designing multi-product biopharmaceutical facilities using evolutionary algorithms. Comput Aid Chem Eng.

[b12] Simaria AS, Gao Y, Turner R, Farid SS (2012). A multi-level meta-heuristic algorithm for the optimization of antibody purification processes. Biochem Eng J.

[b13] Holland JH (1975). Adaptation in Natural and Artificial Systems.

[b14] Box GEP (1957). Evolutionary operation: a method for increasing industrial productivity. Appl Statist.

[b15] Knowles J (2009). Closed-loop evolutionary multiobjective optimization. IEEE Comput Intell Mag.

[b16] Allmendinger R (2012). Tuning evolutionary search for closed-loop optimization.

[b17] Rechenberg I (2000). Case studies in evolutionary experimentation and computation. Comput Methods Appl Mech Eng.

[b18] Judson RS, Rabitz H (1992). Teaching lasers to control molecules. Phys Rev Lett.

[b19] Caschera F, Gazzola G, Bedau MA, Moreno CB, Buchanan A, Cawse J, Packard N, Hanczyc MM (2010). Automated discovery of novel drug formulations using predictive iterated high throughput experimentation. PloS ONE.

[b20] Small BG, McColl BW, Allmendinger R, Pahle J, López-Castejón G, Rothwell NJ, Knowles J, Mendes P, Brough D, Kell DB (2011). Efficient discovery of anti-inflammatory small molecule combinations using evolutionary computing. Nature Chem Biol.

[b21] Vaidyanathan S, Broadhurst DI, Kell DB, Goodacre R (2003). Explanatory optimization of protein mass spectrometry via genetic search. Analyt Chem.

[b22] O'Hagan S, Dunn WB, Brown M, Knowles J, Kell DB (2005). Closed-loop, multiobjective optimization of analytical instrumentation: gas chromatography/time-of-flight mass spectrometry of the metabolomes of human serum and of yeast fermentations. Analyt Chem.

[b23] Weuster-Botz D, Pramatarova V, Spassov G, Wandrey C (1995). Use of a genetic algorithm in the development of a synthetic growth medium for *Arthrobacter simplex* with high hydrocortisone Δ^1^-dehydrogenase activity. J Chem Technol Biotechnol.

[b24] Sivapathasekaran C, Sen R (2013). Performance evaluation of an ANN–GA aided experimental modeling and optimization procedure for enhanced synthesis of marine biosurfactant in a stirred tank reactor. J Chem Technol Biotechnol.

[b25] Low KH, Sørensen E (2005). Simultaneous optimal configuration, design and operation of batch distillation. AIChE J.

[b26] George ED, Farid SS (2008). Stochastic combinatorial optimisation approach to biopharmaceutical portfolio management. Ind Eng Chem Res.

[b27] George ED, Farid SS (2008). Strategic biopharmaceutical portfolio development: an analysis of constraint-induced implications. Biotechnol Prog.

[b28] Farid SS, Washbrook J, Titchener-Hooker NJ (2007). Modelling biopharmaceutical manufacture: design and implementation of SimBiopharma. Comput Chem Eng.

[b29] Friedman M (1937). The use of ranks to avoid the assumption of normality implicit in the analysis of variance. J Am Stat Assoc.

[b30] Hebbron T, Bullock S, Cliff D (2008). Non-uniform epistatic interactions in an extended *NK* model. in Artificial Life.

